# Liver Elastography for Liver Fibrosis Stratification: A Comparison of Three Techniques in a Biopsy-Controlled MASLD Cohort

**DOI:** 10.3390/biomedicines13010138

**Published:** 2025-01-09

**Authors:** Antonio Liguori, Giorgio Esposto, Maria Elena Ainora, Irene Mignini, Raffaele Borriello, Linda Galasso, Mattia Paratore, Maria Cristina Giustiniani, Laura Riccardi, Matteo Garcovich, Antonio Gasbarrini, Luca Miele, Maria Assunta Zocco

**Affiliations:** 1 Centro Malattie Apparato Digerente—CEMAD, Fondazione Policlinico Universitario A. Gemelli, IRCCS, 00168 Roma, Italy; antonio.liguori@guest.policlinicogemelli.it (A.L.); giorgio.esposto@guest.policlinicogemelli.it (G.E.); mariaelena.ainora@policlinicogemelli.it (M.E.A.); irene.mignini@guest.policlinicogemelli.it (I.M.); raffareleborr@gmail.com (R.B.); linda.galasso@guest.policlinicogemelli.it (L.G.); mattia.paratore@guest.policlinicogemelli.it (M.P.); laura.riccardi@policlinicogemelli.it (L.R.); matteo.garcovich@policlinicogemelli.it (M.G.); antonio.gasbarrini@unicatt.it (A.G.); 2 Unità di Medicina Interna e Trapianto di Fegato, Fondazione Policlinico Universitario A. Gemelli, IRCCS, 00168 Roma, Italy; 3 Dipartimento di Scienze Della Salute Della Donna, del Bambino e di Sanità Pubblica, Fondazione Policlinico Universitario A. Gemelli, IRCCS, 00168 Roma, Italy; mariacristina.giustiniani@policlinicogemelli.it; 4 Dipartimento di Scienze Mediche e Chirurgiche, Fondazione Policlinico Gemelli, IRCCS, Università Cattolica del S. Cuore, 8, Largo Gemelli, 00168 Roma, Italy

**Keywords:** SWE, Shearwave elastography, liver fibrosis, MASLD

## Abstract

**Background:** The aim of this study was to investigate the accuracy in fibrosis staging of a novel shear wave elastography (SWE) device (S-Shearwave Imaging by Samsung) and a previously validated 2D-SWE by Supersonic Imagine (SSI) in patients with biopsy proven metabolic dysfunction-associated steatotic liver disease (MASLD). **Methods:** This prospective study included 75 consecutive patients with MASLD who underwent liver biopsy for suspected MASH. All patients underwent S-Shearwave Imaging by Samsung and 2D-SWE with SSI on the same day of liver biopsy. Fibrosis was histologically assessed using the METAVIR classification system. Agreement between the equipment was assessed with the Pearson coefficient. A receiver operator characteristic curve (ROC) analysis with the Youden index was used to establish thresholds for fibrosis staging. **Results:** A good correlation was found between S-Shearwave Imaging by Samsung and 2D-SWE with SSI (Pearson’s R = 0.68; *p* < 0.01). At multivariate regression analysis, S-Shearwave Imaging was associated with advanced fibrosis (≥F3) independently from age, diabetes and platelets (OR 2.94, CI 1.69–5.11, *p* < 0.01). The fibrosis diagnostic accuracy of both S-Shearwave Imaging and 2D-SWE was good to optimal with AUROCs of 0.81 and 0.70 for significant fibrosis (≥F2), 0.94 and 0.91 for severe fibrosis (≥F3), respectively. The accuracy of S-Shearwave is not significantly different from Fibroscan and Agile3+ (DeLong test *p* value 0.16 and 0.15, respectively) while is slightly better than 2D-SWE, FIB4 and NFS (DeLong test *p* value < 0.05). For S-Shearwave Imaging by Samsung, the best cut-off values for diagnosing fibrosis ≥F2, ≥F3 were, respectively, 7.9 kPa (Sens 74.4%, Spec 87.5%) and 8.1 kPa (Sens 95.6%, Spec 78.8%). For 2D-SWE by SSI, the best cut-off values for diagnosing fibrosis ≥F2, ≥F3 were, respectively, 7.2 kPa (Sens 55.8%, Spec 84.4%) and 7.6 kPa (Sens 82.6%, Spec 84.6%). **Conclusion:** S-Shearwave Imaging is a useful and reliable non-invasive technique for staging liver fibrosis in patients with MASLD. Its diagnostic accuracy is non-inferior to other shear wave elastography techniques (TE and 2D-SWE by SSI).

## 1. Introduction

Metabolic dysfunction-associated steatotic liver disease (MASLD) is by far the most common liver disease, with an estimated global prevalence of 25% [1]. MASLD, defined by increased liver fat storage without significant alcohol consumption or other causes of liver disease, ranges from simple steatosis to progressive steatohepatitis (MASH), and further on to cirrhosis. The quantification of liver stiffness by ultrasound elastography (UE) is pivotal in staging fibrosis, guiding treatment strategies, and monitoring disease progression. Among UE technologies, transient elastography (TE) (FibroScan, Echosens, Paris, France) is the one that best correlates with the stage of portal hypertension [2] and histological fibrosis [3,4]. The main limitations of TE are the inability to choose the region of interest (ROI), and therefore to avoid blood vessels and liver lesions, and the necessity of a dedicated instrument. To overcome these limits, UE has been implemented developing technologies directly integrated into ultrasound machines, like 2D shear wave elastography (2D-SWE), which allows a two-dimensional evaluation of liver stiffness with the advantage of a broader area of interest chosen in real time by ultrasound B-mode images. The physician is therefore able to select the best ROI and sample multiple liver areas in real time while conducting the standard ultrasound exam. Among these new techniques, Samsung (Medison Co., Ltd., Seoul, Republic of Korea) has recently introduced a novel 2D-SWE technology, named S-Shearwave elastography. The reliability of liver stiffness measurements (LSMs) obtained by S-Shearwave is granted by an index (Reliability Measurement Index, RMI) calculated by the weighted sum of the magnitude of the shear wave and the residual of the wave equation. Therefore, the higher RMI correlates with reliable LSMs, allowing for the assessment of the quality of data produced by S-Shearwave. Intra- and interobserver agreements were prospectively validated, and data revealed an excellent repeatability [5,6]. The validity of S-Shearwave compared to TE and liver biopsy was initially explored by Yoo HW et al. in 2022 [7] in a prospective study on patients with chronic liver disease who underwent S-Shearwave, TE and liver biopsy. The results of S-Shearwave statistically correlated with the stages of histological fibrosis (r = 0.601, *p* < 0.001), proving a good diagnostic validity (AUROC, 0.851; 95% CI, 0.773 to 0.911) for the diagnosis of fibrosis (≥F2), with a cut-off value of 5.8 kPa, and for the diagnosis of liver cirrhosis (AUROC 0.889; 95% CI, 0.817 to 0.940) with a cut-off value of 9.6 kPa. As these preliminary results proved that S-Shearwave elastography is reliable compared to liver histology and TE, further studies are needed to extensively validate the technique [7,8].

Whereas US elastography plays a key role in the identification of LSMs [8], it is also known that the different etiology of liver disease, and, consequently, the different mechanisms of fibrogenesis, leads to a different interpretation of LSMs [9,10]. Indeed, European and American guidelines outline pathology-specific cut-offs to identify patients with advanced fibrosis [11,12]. Moreover, the interpretation of LSMs should account for comorbidities like myeloproliferative neoplasms (MPNs), which could increase spleen and liver stiffness, whose values been linked to bone marrow fibrosis [13]. In this context, the application of liver and spleen shear wave elastography represents a novel approach to assess treatment response and prognosis in MPNs [13].

Furthermore, these guidelines highlight that TE is the most validated method, while they encourage validation studies of other UE techniques in order to expand the use of UE and improve the management of patients with chronic liver disease.

The aim of our study is to investigate the accuracy in the fibrosis staging of S-Shearwave Imaging by Samsung compared to previously validated 2D-SWE by Supersonic Imagine (SSI) in patients with a histopathological diagnosis of MASLD.

## 2. Materials and Methods

This is a monocentric cross-sectional study performed during a 1-year interval (January 2021 to December 2021) in a tertiary Department of Gastroenterology and Hepatology in Italy. All consecutive patients with a clinical suspicion of MASLD/MASH, referred to the Liver Disease Unit of the Fondazione Policlinico Universitario A. Gemelli in Rome for percutaneous liver biopsy, were enrolled in this study. The indication for liver biopsy was the US evidence of fatty liver and deranged liver function tests. The diagnosis of MASLD was based on the recent multi-society Delphi consensus [14].

Inclusion criteria were an age > 18 years, liver steatosis documented by abdominal ultrasound, a platelet count of at least 80 × 10^9^/L, a prothrombin time international normalized ratio of less than 1.5 and signed informed consent. Subjects with increased alcohol consumption (ethanol intake >3 alcohol units per day for men and >2 alcohol units per day for women) were excluded. Other exclusion criteria were chronic liver disease of clear etiology (autoimmune hepatitis, primary biliary cholangitis or primary sclerosing cholangitis, hepatitis C virus infection, hepatitis B virus infection), ascites or other clinical signs of portal hypertension (gastro-esophageal varices, hepatic encephalopathy, porto-systemic shunts), biliary obstruction and oncologic history.

### 2.1. Study Design

On the same date of liver biopsy, all patients underwent US-based measurements of liver stiffness using 3 distinctive systems: 2D-SWE was performed using the Aixplorer MACH 30 system (SuperSonic Imagine, Aix-en-Provence, France), transient elastography (TE) was performed with the FibroScan system (EchoSens, Paris, France), S-Shearwave elastography was performed with RS85 Prestige (Samsung, Seoul, Republic of Korea). The same day, height and weight were derived from each patient to calculate body mass index (BMI), and blood samples were obtained for the evaluation of complete blood counts, thrombocytes, total cholesterol, triglycerides, aminotransferase levels, gamma-glutamyltransferase (GGT) and albumin. The fibrosis 4 score (FIB-4) [15], NAFLD fibrosis score (NFS) [16] and Agile3+ [17] score were calculated for each patient. Finally, comorbidities (diabetes mellitus, arterial hypertension) were collected from medical records.

The study protocol was approved by the Ethical Review Board of Fondazione Policlinico Universitario “A. Gemelli” IRCCS and conformed to the ethical guidelines of the Declaration of Helsinki. All patients provided written informed consent before inclusion. 

The primary outcome of this study was to determine the diagnostic performance of different US-based techniques for the non-invasive assessment of liver fibrosis compared to histologic evaluation.

### 2.2. Two-Dimensional Shear Wave Elastography

Two-dimensional SWE was carried out with the Aixplorer MACH 30 system (SuperSonic Imagine, Aix-en-Provence, France), equipped with a wideband C1-6 MHz curvilinear probe, by two highly trained operators (M.E.A and A.L., respectively, with 12 and 6-years of experience in liver US), both not aware of the clinical data and the results of the histopathologic assessment. Each examination was performed twice, one by each operator, and the mean value was considered for the analysis. According to EFSUMB and WFUMB guidelines, all patients fasted more than 8 h and were placed supine with the right arm in maximal abduction [18,19]. First, we performed B-mode scanning by an intercostal approach, to obtain a suitable image and select the best acoustic window according to a standardized protocol. The LS evaluation was performed with the shear wave technique by positioning the measurement box at least 1.5 cm below the liver capsule, avoiding large vessels or bile ducts and rib shadows. While the patient holds their breath, measurements were obtained selecting a 15 mm diameter region of interest (ROI) in a color map box with complete and homogeneous filling. Each SWE measurement was acquired aiming at a stability index of at least 90% to guarantee both spatial and temporal stability within the circular ROI. The median value of 3 successful measurements was then obtained from each patient with an interquartile range to the median ratio (IQR/M) <30% as a measurement reliability criterion. The results were expressed in kilopascals (kPa).

### 2.3. Transient Elastography

TE measurements were performed with the FibroScan Compact 530 system equipped with the M (3.5 Hz frequency) probe or the XL (2.5 Hz frequency) probe. All patients were evaluated in fasting conditions (at least 6 h) on the same day of liver biopsy and multiparametric US examination by a single qualified operator (A.L. with 8 years of experience in TE), according to the EFSUMB and WFUMB guidelines [18,19]. The operator was blinded to histopathologic results. Results were considered reliable if the median value of 10 valid measurements reported an IQR/M < 30%, expressed in kPa for fibrosis (range 2.5–75 kPa).

### 2.4. S-Shearwave Elastography

Two-dimensional SWE examination for liver stiffness measurement was performed using the S-Shearwave Imaging application on the RS85 Ultrasound system equipped with a convex array CA1-7A transducer (Samsung Medison Co., Ltd., Seoul, Republic of Korea). Measures were conducted by two trained operators (M.E.A and A.L., respectively, with 12 and 6 years of experience in liver US), blinded to both the clinical data and the results of histopathologic assessment. Each examination was performed twice, one by each operator, and the mean value was used for the analysis. Patients were prepared according to WFUMB and EFSUMB guidelines, as described above. The LS evaluation was performed with the 2D-SWE technique by positioning the shear wave measurement box at least 1.5 cm below the liver capsule, avoiding large vessels or bile ducts and rib shadows. Measurements were obtained through a 10 mm diameter ROI in the center of a color map with a homogenous color pattern and reliable measurement index (RMI) map above 0.3, to guarantee the spatial and temporal stiffness stability within the circular ROI. The median value of 3 successful LS measurements was obtained from each patient with an interquartile range to the median ratio (IQR/M) <30% as a measurement reliability criterion. The results were expressed in kilopascals (kPa).

### 2.5. Liver Biopsy and Histopathologic Evaluation

Liver biopsy was performed right after imaging procedures under real-time US guidance using a 17- or 18-gauge core needle biopsy kit at a location as close as possible to the area in which US examination was performed. Tissue specimens were fixed in formalin and stained with hematoxylin–eosin and Masson trichrome. All liver specimens were examined by two senior pathologists with more than 15 years of experience in hepatic pathologic assessment, unaware of the results of imaging techniques. The degree of hepatic steatosis, hepatocyte ballooning, lobular inflammatory activity and the liver fibrosis stage was assessed according to the histologic scoring system for MASLD [20]. Steatosis was graded on a scale of 0 to 3 (S0–S3), hepatocyte ballooning on a scale of 0 to 2 (B0–B2), lobular inflammatory activity on a scale of 0 to 3 (I0–I3) and the fibrosis stage on a scale of 0 to 4 (F0–F4). After the assessment of each element, the NAFLD activity score (NAS) was also calculated. Significant fibrosis was defined as Fibrosis ≥ F2 while advanced fibrosis was defined as Fibrosis ≥ F3.

### 2.6. Statistical Analysis

Descriptive statistics were used for demographic, anthropometric, elastographic and laboratory findings. Numerical variables are presented as means ± standard deviation (in case of normal distribution) or the median value and 1st–3rd quartiles (in case of non-normal distribution). Categorical variables were presented as numbers and percentages. Parametric tests (t-test or ANOVA) were used for the assessment of differences between numerical variables with normal distribution, while a non-parametric test (Kruskal–Wallis) was used for variables with non-normal distribution. A chi-square test was used to determine significant differences between categorical variables. Pearson and Spearman linear correlation coefficients were used to evaluate the relationship between continuous variables, respectively, when normality was assessed or not. The overall diagnostic performance of the imaging parameters was estimated according to the area under the receiver operating characteristic curve (AUROC) together with the 95% confidence interval (CI). The comparison of AUROCs was conducted with the Delong test. The cut-off values were set to maximize the Youden index for staging liver fibrosis as compared to histopathology scores, which were used as the reference method. For these cut-off values, the sensitivity, specificity, positive predictive value (PPV) and negative predictive value (NPV) were reported. We used univariable and multivariable logistic regression analyses to determine significant determinant factors for advanced fibrosis. All variables with *p* < 0.05 in the univariable analysis were included in the multivariable analysis. All tests were considered statistically significant in the case of *p* < 0.05. The statistical analysis was performed using SPSS 20.0, R (R 4.4.2) and STATA14 (STATA 18) software, and the statistical significance was defined as a *p* value below 0.05.

## 3. Results

### 3.1. Clinical, Histological, and Ultrasound Characteristics of the Study Population

Seventy-five patients were included in this study, and the main characteristics are presented in Table 1. A total of 42 (56%) patients were male, the median age was 52 years and the median BMI was 31.1 kg/m2. The prevalence of obesity, diabetes and hypertension was 54%, 28% and 40%, respectively. The median AST, ALT and GGT were, respectively, 34 (IQR 24–50) IU/L, 51 (IQR 29–72) IU/L and 65 (IQR 29–100) IU/L. According to histopathological assessment, 20 (26.7%) patients had severe steatosis (S3), 63 (94.6%) patients had a ballooning grade ≥1, 23 (30.6%) patients had advanced fibrosis (F ≥ 3) and 50 (66.6%) patients had MASH. Median liver stiffness (LSM) with S-Shearwave, 2D-SWE and Fibroscan was 7.7 kPa, 6.5 kPa and 6.7 kPa, respectively. Patients were stratified according to the presence of advanced fibrosis (≥F3) (Table 1). The prevalence of diabetes and hypertension was significantly higher in patients with advanced fibrosis (47.8% and 73.9%, respectively) compared with patients without advanced fibrosis (19.2% and 25%, respectively) (*p* = 0.01). Patients with advanced fibrosis had a significantly higher median age (54 vs. 50 years, *p* = 0.01) and significantly lower median platelet count (198 vs. 239 × 10^6^/mL, *p* = 0.01), median albumin level (38 vs. 42 g/L, *p* < 0.01) and median total cholesterol level (184 vs. 204 mg/dL, *p* = 0.02). Median LSMs were significantly higher in the advanced fibrosis group both with transient elastography (10.7 vs. 5.8 kPa, *p* < 0.01), S-Shearwave (11.1 vs. 6.6 kPa, *p* < 0.01) and 2D-SWE (9.4 vs. 5.7, *p* < 0.01). Median FIB4, NFS and Agile3+ were significantly higher in the advanced fibrosis group (1.52, 0.20 and 0.67, respectively) compared with patients without advanced fibrosis (0.92, −1.96, 0.09, respectively) (*p* < 0.05).

### 3.2. Relationship Between S-Shearwave Elastography, 2D-Shear Wave Elastography and Transient Elastography and Association to Histological Liver Fibrosis

**Figure 1:**
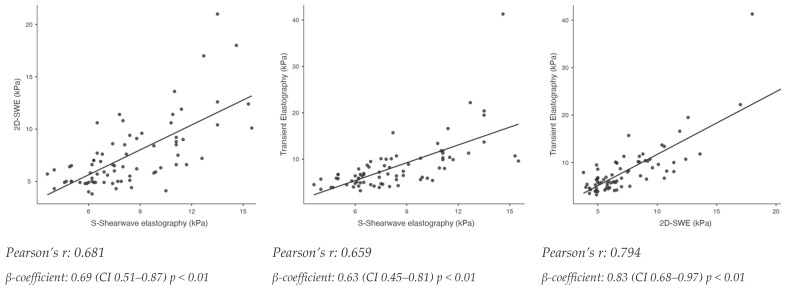
Correlation matrix showing relationship between S-Shearwave elastography, 2D-shear wave elastography and transient elastography. Scatterplots showing linear relationship between S-Shearwave, 2D-SWE and transient elastography. Pearson’s r and linear regression β-coefficient are shown. Multivariable adjustments considering ALT and BMI are conducted for linear regression models.

Correlation between S-Shearwave, 2D-SWE and TE is shown in Figure 1. The highest Pearson’s correlation coefficient (r) was found between TE and 2D-SWE (r = 0.794, *p* < 0.01). Pearson’s correlation coefficient (r) between S-Shearwave elastography and TE was 0.659 (*p* < 0.01), while Pearson’s correlation coefficient (r) between S-Shearwave elastography and 2D-SWE was 0.681 (*p* < 0.01).

For the univariate regression analysis, age, total cholesterol, platelets, diabetes, hypertension, S-Shearwave elastography, 2D-SWE and LSM by TE were significant factors associated with advanced fibrosis diagnosis at the histopathological assessment (*p* < 0.05, Table 2). Focusing on S-Shearwave elastography, multivariate analysis revealed that it is a significant predictor of advanced liver fibrosis independently from age, platelet count and diabetes (OR 2.94, CI 1.69–5.11, *p* < 0.01).

**Figure 2:**
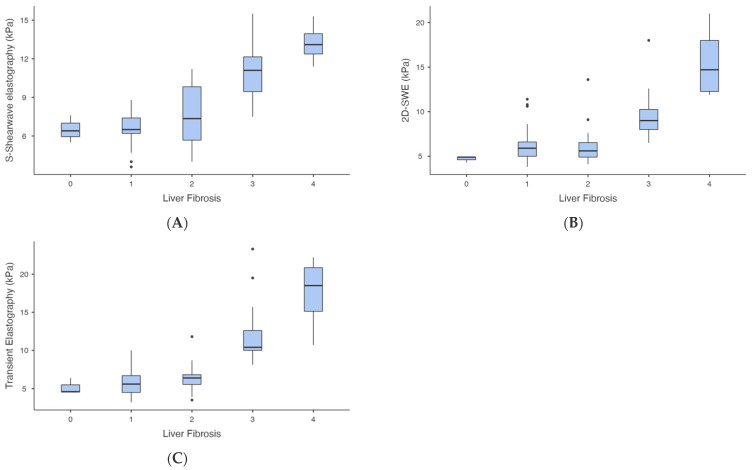
Distribution of liver stiffness measurements (Fibroscan, S-Shearwave and 2D-SWE) according to fibrosis stage. Box plots showing distribution of liver stiffness measurements by S-Shearwave elastography (**A**), 2D-SWE (**B**) and Fibroscan (**C**) according to fibrosis stage at histopathologic assessment.

LSM by S-Shearwave elastography increased as the degree of liver fibrosis increased (*p* < 0.01 from one-way ANOVA), as well as 2D-SWE and TE (*p* < 0.01 from one-way ANOVA) (Figure 2). There was a significant difference in the LSM by S-Shearwave elastography between each group of different degrees of liver fibrosis (*p*  <  0.05), except for between the patients with F1 and F0 (*p* = 1.00), F2 and F1 (*p* = 0.69) and F4 and F3 (*p* = 0.33).

There was a significant difference in the LSM by TE between each group of different degrees of liver fibrosis (*p*  <  0.05), except between the patients with F1 and F0 (*p* = 0.98), F2 and F1 (*p* = 0.74) and F4 and F3 (*p* = 0.23). There was a significant difference in the LSM by 2D-SWE between each group of different degrees of liver fibrosis (*p*  <  0.05), except for between the patients with F1 and F0 (*p* = 0.25) and F2 and F1 (*p* = 0.95).

### 3.3. Performances of S-Shearwave Elastography, 2D-Shear Wave Elastography and Transient Elastography for Detection of Significant Fibrosis (F ≥ 2) and Advanced Fibrosis (F ≥ 3)

**Figure 3:**
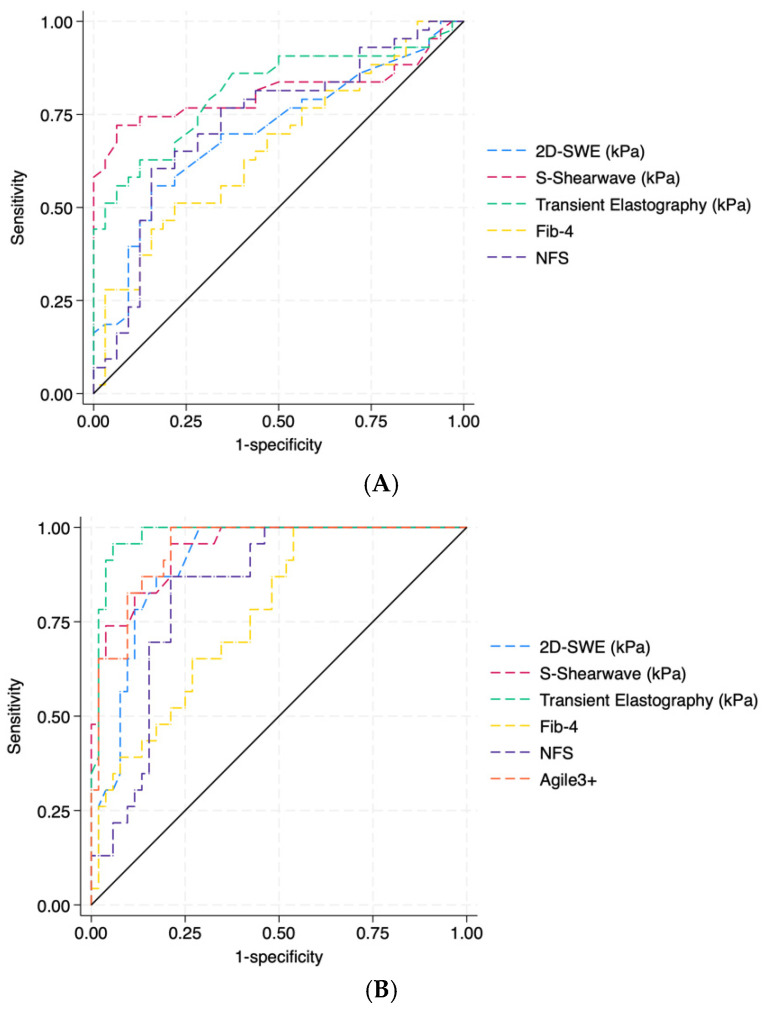
Performance of non-invasive diagnostic tests (TE, 2D-SWE, S-Shearwave by Samsung, NFS, Agile3+ and FIB-4) for predicting significant fibrosis (F ≥ 2, (**A**)) and advanced fibrosis (F ≥ 3, (**B**)). TE = transient elastography; 2D-SWE = two-dimensional shear wave elastography; FIB-4 = fibrosis score; and NFS = nonalcoholic fatty liver disease fibrosis score.

The AUC for the detection of significant fibrosis (F ≥ 2) was good for FIB4 (0.703), NFS (0.661) and 2D-SWE (0.729), and it was very good for S-Shearwave (0.810) and TE (0.813) (Figure 3). The accuracy of S-Shearwave was not significantly different from TE’s accuracy (DeLong test *p* value = 0.95) while slightly better than 2D-SWE, FIB4 and NFS (DeLong test *p* value < 0.05). The best cut-off identified according to the maximum Youden Index for S-Shearwave elastography for the diagnosis of significant fibrosis is 7.9 kPa with a sensitivity and specificity of 74.4% and 87.5%, respectively (Table 3). The best cut-off identified by Fibroscan and 2D-SWE for the diagnosis of significant fibrosis is 8.0 kPa (Sens 62.7%, Spec 97.5%) and 7.2 kPa (Sens 55.8% and Spec 84.4%), respectively.

The AUC for the detection of advanced fibrosis (F ≥ 3) was good for FIB4 (0.768) and was very good to excellent for S-Shearwave (0.942), TE (0.979), 2D-SWE (0.906), NFS (0.833) and Agile3+ (0.944) (Figure 3). The accuracy of S-Shearwave was not significantly different from Fibroscan and Agile3+ (DeLong test *p* value 0.16 and 0.15, respectively) while slightly better than 2D-SWE, FIB4 and NFS (DeLong test *p* value <0.05). The best cut-off identified according to the maximum Youden Index for S-Shearwave elastography for the diagnosis of advanced fibrosis is 8.1 kPa with a sensitivity and specificity of 95.6% and 78.8%, respectively (Table 3). The best cut-off identified for TE and 2D-SWE for the diagnosis of advanced fibrosis is 8.9kPa (Sens 95.6%, Spec 94.2%) and 7.6kPa (Sens 82.6% and Spec 84.6%), respectively. Using the EASL guidelines’ cut-offs for TE, the sensitivity of low cut-off (8 kPa) was 100%, and the specificity of high cut-off (12 kPa) was 98.0%. Using the EASL guidelines’ cut-offs for Agile 3+, the sensitivity of low cut-off (0.451 kPa) was 82.6%, and the specificity of high cut-off (0.679 kPa) was 98.8%.11

## 4. Discussion

Our study showed that S-Shearwave elastography is a reliable method for predicting the stage of liver fibrosis in MASLD patients and that it is non-inferior to other 2D-SWE method (Supersonic Imagine) and to TE. One of the main advantages of S-Shearwave elastography compared to other elastography techniques is the real-time map that displays the Reliable Measurement Index (RMI), allowing for the selection of an ROI that ensures greater reliability of the result. Previous studies have shown that both the intra- and inter-observer repeatability of LSMs obtained using the S-Shearwave Imaging technique in patients with chronic liver disease were excellent (intraclass correlation coefficients 0.997 and 0.995, respectively) [6]. This enhances its reliability, as well as the repeatability and comparability of results. S-Shearwave elastography shows its best performance in differentiating the more advanced stages of fibrosis (F ≥ 3) with an AUC of 0.942. The optimal cut-off value for S-Shearwave elastography to diagnose advanced fibrosis is 8.1 kPa, which provides high sensitivity (95.6%) and good specificity (78.8%). A previous study assessed the performance of S-Shearwave elastography in stratifying liver fibrosis in a cohort of patients with chronic liver disease of various etiologies, including MASLD (30% of patients) [7]. The results of the study are comparable to ours, with an AUC of 0.917 for the diagnosis of advanced fibrosis and a proposed cut-off of 7.55 kPa, which provided sensitivity and specificity in line with those shown in our study (Sens 95.5%, Spec 81.7%). 

The performance of S-Shearwave in diagnosing significant fibrosis (≥F2) was excellent in our study (AUC 0.810), similar to what was reported by Yoo HW et al. (AUROC, 0.851) [7]. The optimal cut-off in our study is higher than in Yoo HW et al.’s study (7.9 kPa vs. 5.8 kPa). This is reflected in the higher specificity (87.5% vs. 74.4%) and lower sensitivity (74.4% vs. 88.9%) of the cut-off that we propose. The choice of cut-off to apply in clinical practice depends on the aim, and, often, a dual-cut-off strategy is preferred. This approach involves selecting a lower cut-off with high sensitivity to identify patients at low risk of significant fibrosis and a higher cut-off with high specificity to identify patients at high risk of significant fibrosis. The sample size of our study does not allow for a reliable analysis with two cut-offs, but the difference in sensitivity and specificity between our proposed cut-off and that of Yoo HW et al. suggests that this approach should be pursued in multicenter studies with larger cohorts.

Several studies have assessed the diagnostic performance of 2D-SWE measured with devices produced by different companies, and the results are consistent with those highlighted in our study. Recent meta-analyses have indeed shown the AUROC, sensitivities, and specificities in the 0.80–1.0 range for all US-based methods when estimating either significant or advanced fibrosis [21,22,23].

Our study demonstrates a moderate correlation between S-Shearwave elastography and TE (Pearson’s r = 0.659) in the MASLD population and comparable performance between the two methods in diagnosing significant fibrosis (≥F2) and advanced fibrosis (≥F3) (De Long test *p* > 0.05), using liver biopsy as the gold standard. In our study, 2D-SWE showed a moderate correlation with S-Shearwave and TE (Pearson’s r: 0.681 and 0.794, respectively), but its diagnostic performance for significant fibrosis (≥F2) and advanced fibrosis (≥F3) was lower than that of TE (De Long test *p* < 0.05). However, it is important to note that, although both methods measure the same viscoelastic property of the tissue in the same unit (kPa), the numerical results are not directly comparable. To achieve the same sensitivity or specificity in diagnosing significant (≥F2) and advanced fibrosis (≥F3), the cut-offs for TE are higher than those for S-Shearwave elastography. This supports the findings of large meta-analyses and guidelines, which indicate that a single LSM value should be interpreted based on the specific tool used and cannot be compared across different methods or between different devices using the same 2D-SWE technique.

The strengths of our study lie in its methodology. Firstly, patients were consecutively enrolled over a short period of time. All patients underwent the various elastography tests and blood tests needed to calculate FIB4, NFS and Agile3+ on the same day, which coincided with the biopsy date. This ensures maximum comparability between the results of non-invasive tests and with the histological findings. Another advantage of our study is the balanced distribution of fibrosis stages, particularly the intermediate stages (F1, F2, and F3), allowing a more reliable assessment of the discriminatory power of non-invasive tests. The main limitation of our study is its single-center design, which does not allow for a large sample size. A larger sample would certainly make the study results more reliable and broadly applicable. Another limitation is the absence of a healthy control group that could affect the lower threshold between normal and pathological liver stiffness.

## 5. Conclusions

In conclusion, S-Shearwave Imaging technology and 2D-SWE are reliable for the non-invasive evaluation of liver fibrosis severity in patients with MASLD, with performance comparable to that of TE.

## Figures and Tables

**Table 1:** Demographic, clinical, histological, elastographic and biochemical characteristics of the enrolled patients, stratified according to advanced fibrosis diagnosis at liver biopsy (F ≥ 3).

	**Entire Cohort**	**F < 3**	**F ≥ 3**	*p* Value *
	***n* = 75**	***n* = 52**	***n* = 23**	
Gender (male)	42 (56%)	31 (59.6%)	11 (47.8%)	0.34
Age (years)	52 (44–56)	50 (39–55)	54 (51–58)	0.01
BMI (kg/m^2^)	31.1 (28.1–37.8)	30.4 (27.7–35.4)	33.6 (29.1–40.7)	0.07
Obesity	41 (54.7%)	26 (50%)	15 (65.2%)	0.22
Diabetes	21 (28%)	10 (19.2%)	11 (47.8%)	0.01
Hypertension	30 (40%)	13 (25%)	17 (73.9%)	<0.01
Biochemical Profile				
Platelet count (×10^6^/mL)	233 (187–268)	239 (202–281)	198 (165–238)	<0.01
AST (IU/L)	34 (24–50)	33 (24–45)	42 (24–52)	0.94
ALT (IU/L)	51 (29–72)	51 (28–75)	51 (30–61)	0.47
GGT (IU/L)	65 (29–100)	47 (30–92)	66 (30–124)	0.85
ALP (IU/L)	66 (53–88)	64 (41–87)	70 (57–89)	0.95
Albumin (g/L)	41 (38–43)	42 (41–43)	38 (37–39)	<0.01
Triglycerides (mg/dL)	140 (93–89)	142 (96–189)	133 (90–187)	0.92
Total Cholesterol (mg/dL)	199 (173–221)	204 (177–229)	184 (165–208)	0.02
HDL (mg/dL)	45 (33–50)	45 (33–50)	40 (34–49)	0.20
FIB4	1.12 (0.78–1.77)	0.92 (0.73–1.37)	1.52 (1.10–2.32)	0.03
NFS	−1.31 (−2.59–0.20)	−1.96 (−3.01–−1.18)	0.20 (−0.85–0.84)	<0.01
Agile3+	0.17 (0.06–0.57)	0.09 (0.05–0.21)	0.67 (0.52–0.79)	<0.01
Histologic Findings				
Steatosis Grade				0.45
1	30 (40%)	23 (44.2%)	7 (30.4%)	
2	25 (33.3%)	17 (32.7%)	8 (34.7%)	
3	20 (26.7%)	12 (23.1%)	8 (34.7%)	
Lobular Inflammation Grade				0.21
0	4 (5.3%)	4 (7.7%)	0	
1	55 (73.3%)	39 (75%)	16 (69.6%)	
2	16 (21.3%)	9 (17.3%)	7 (30.4%)	
3	0	0	0	
Hepatocyte Ballooning Grade				0.03
0	12 (16%)	12 (23.1%)	0	
1	54 (72%)	35 (67.3%)	19 (82.6%)	
2	9 (12%)	5 (9.6%)	4 (17.4%)	
Fibrosis Stage				<0.01
0	3 (4%)	3 (5.7%)	0	
1	29 (38.7%)	29 (38.4%)	0	
2	20 (26.7%)	20 (38.5%)	0	
3	19 (25.3%)	0	19 (82.6%)	
4	4 (5.3%)	0	4 (17.4%)	
MASH	50 (66.6%)	30 (57.7%)	20 (86.9%)	0.01
Elastographic measurements				
LSM by TE (kPa)	6.7 (5.2–10.1)	5.8 (4.6–6.7)	10.7 (10.1–14.7)	<0.01
S-Shearwave Samsung (kPa)	7.7 (6.25–10.7)	6.6 (6.0–7.9)	11.1 (10.3–13.1)	<0.01
2D-SWE (kPa)	6.5 (5.0–8.9)	5.7 (4.9–6.6)	9.4 (8.5–11.6)	<0.01
CAP (dB/m)	317 (259–370)	316 (244–356)	340 (277–380)	0.06
Att.PLUS (dB/cm/mHz)	0.50 (0.42–0.61)	0.49 (0.42–0.63)	0.54 (0.44–0.59)	0.87
SSP.PLUS (m/s)	1535 (1508–1565)	1537 (1500–1570)	1520 (1510–1552)	0.60
TAI (dB/cm/MHz)	0.93 (0.80–1.06)	0.93 (0.75–1.04)	0.91 (0.82–1.10)	0.86
TSI	96.7 (91.0–102.0)	96.8 (90.7–102.4)	96.3 (91.3–102.1)	0.60

Data are presented as number and percentage or median (interquartile range). BMI = body mass index; AST = aspartate aminotransferase; ALT = alanine aminotransferase; GGT = gamma-glutamyl transpeptidase; FIB-4 = fibrosis score; NFS = nonalcoholic fatty liver disease fibrosis score; MASH = metabolic dysfunction-associated steatohepatitis; LSM = liver stiffness measurement; TE = transient elastography; 2D-SWE = two-dimensional shear wave elastography; CAP = controlled attenuation parameter; Att.PLUS = attenuation plane-wave ultrasound; SSp.PLUS = sound speed plane-wave ultrasound; tai = tissue attenuation imaging; and tsi = tissue scatter distribution imaging. * *p* value of non-parametric test (Kruskal–Wallis) for continuous variables, and chi-square test for categorical variables.

**Table 2:** Univariate and multivariate analysis evaluating association between S-Shearwave by Samsung and advanced fibrosis (F ≥ 3).

Variable	Univariate Analysis		Multivariate Analysis	
	OR (CI 95%)	*p* Value	OR (CI 95%)	*p* Value
Sex (male)	0.62 (0.23–1.67)	0.34		
Age	1.07 (1.01–1.14)	0.02	1.05 (0.94–1.17)	0.41
Obesity	1.87 (0.68–5.18)	0.22		
Triglycerides	1.00 (0.99–1.01)	0.92		
Total Cholesterol	0.98 (0.97–0.99)	0.03		
Platelets	0.98 (0.97–0.99)	<0.01	0.98 (0.96–1.01)	0.10
Diabetes	3.85 (1.32–11.22)	0.01	3.44 (0.49–24.4)	0.21
Hypertension	8.5 (2.76–26.1)	<0.01		
ALT	0.99 (0.98–1.01)	0.48		
AST	1.00 (0.98–1.01)	0.94		
S-Shearwave Samsung (kPa)	2.71 (1.75–4.20)	<0.01	2.94 (1.69 –5.11)	<0.01
2D-SWE (kPa)	1.90 (1.40–2.58)	<0.01		
LSM by TE (kPa)	4.18 (1.95–8.95)	<0.01		

OR = odds ratio, and CI = confidence interval.

**Table 3:** Performance of non-invasive diagnostic tests (S-Shearwave, 2D-SWE (kPa), Fibroscan, FIB4 and NFS) for predicting significant fibrosis (F ≥ 2) or advanced fibrosis (F ≥ 3).

Variable	Cut-Off Value	AUC (95% CI)	Sensitivity (%)	Specificity (%)	PPV (%)	NPV (%)	De Long *p* Value
**F ≥ 2**							
LS by TE (kPa)	8.0	0.813 (0.715–0.910)	62.7	97.5	87.1	63.4	Ref
S-Shearwave Samsung (kPa)	7.9	0.810 (0.706–0.913)	74.4	87.5	88.9	71.8	0.95
2D-SWE (kPa)	7.2	0.703 (0.584–0.822)	55.8	84.4	82.7	58.7	0.01
FIB4	1.25	0.661 (0.537–0.785)	51.1	78.1	75.8	54.3	0.02
NFS	−0.94	0.729 (0.609–0.848)	60.5	84.4	83.9	61.4	0.13
**F ≥ 3**							
LS by TE (kPa)	8.9	0.979 (0.950–1.000)	95.6	94.2	88.0	98.0	Ref
	8.0		100	84.6	74.2	100	
	12.0		39.1	98.0	90.0	78.4	
S-Shearwave Samsung (kPa)	8.1	0.942 (0.893–0.991)	95.6	78.8	66.6	97.6	0.16
2D-SWE (kPa)	7.6	0.906 (0.839–0.971)	82.6	84.6	70.4	91.6	0.01
FIB4	0.85	0.768 (0.659–0.876)	100	46.1	45.1	100	<0.01
NFS	−0.94	0.833 (0.741–0.924)	86.9	78.8	64.5	93.2	<0.01
Agile3+	0.24	0.944 (0.897–0.991)	100	78.8	67.6	100	0.11
	≤0.451		82.6	86.5	73.1	91.8	
	≥0.679		47.8	98.8	91.6	80.9	

AUROC = area under the receiver operating characteristics curve; CI = confidence interval; PPV = positive predictive value; NPV = negative predictive value; FIB-4 = fibrosis score; NFS = nonalcoholic fatty liver disease fibrosis score; LS = liver stiffness; TE = transient elastography; and 2D-SWE = two-dimensional shear wave elastography.

## Data Availability

Data are contained within the article.
